# Belzutifan in Von Hippel-Lindau Syndrome and Renal Cell Carcinoma: Mechanisms, Clinical Evidence, and Future Directions

**DOI:** 10.7759/cureus.108967

**Published:** 2026-05-16

**Authors:** Jooah Park, Heena Arshad, Sharib Khan, Neha Pillai Vinod, Riya Goel, Divyansh Vats, Inamullah Arshad

**Affiliations:** 1 General Internal Medicine, Gongju Public Health Center, Gongju, KOR; 2 Internal Medicine, Sargodha Medical College, Sargodha, PAK; 3 Internal Medicine, Kasturba Medical College (KMC), Manipal, IND; 4 Internal Medicine, SRM Medical College and Research Center, Chennai, IND; 5 Internal Medicine, Subharti Medical College, Meerut, IND; 6 Internal Medicine, Shalamar Medical and Dental College, Lahore, PAK

**Keywords:** belzutifan, clear cell renal carcinoma, hif-2α inhibition, renal cell carcinoma, targeted cancer therapy, vhl disease, von hippel-lindau syndrome

## Abstract

Clear cell renal carcinoma (ccRCC) and von Hippel-Lindau (VHL)-associated tumors arise due to dysregulation of the VHL/HIF pathway resulting from loss or inactivation of the VHL tumor suppressor gene, leading to constitutive activation of hypoxia-inducible factors (HIFs). Although immune checkpoint inhibitors and VEGF-targeted therapies have improved outcomes, these treatment modalities do not directly inhibit HIF-dependent transcription factors and are limited by resistance, cumulative adverse effects, and incomplete pathway suppression. With the introduction of belzutifan, a selective HIF-2α inhibitor, a remarkable drug has emerged, revolutionizing the management of VHL disease and ccRCC.

Belzutifan directly inhibits HIF-2α, the key oncogenic driver, and represents a paradigm shift in the treatment of VHL-associated tumors and advanced ccRCC. Across multiple clinical studies, it has demonstrated consistent antitumor activity and durable disease control, along with a predictable, mechanism-based safety profile characterized primarily by anemia and hypoxia. This review focuses on belzutifan, with the aim of describing its biological rationale, pharmacological features, and key clinical efficacy and safety data, as well as clarifying its evolving role within current and emerging treatment landscapes.

Remaining challenges include uncertainty regarding the identification of predictive biomarkers, optimization of patient selection, and integration of combination or sequencing strategies. Beyond its immediate clinical impact, belzutifan establishes a foundation for next-generation hypoxia-targeted therapies.

## Introduction and background

Von Hippel-Lindau (VHL) syndrome is a hereditary cancer predisposition condition characterized by dysregulated cellular oxygen sensing and the development of highly vascular tumors across multiple organ systems. Inherited in an autosomal dominant manner, VHL syndrome arises from germline alterations in the VHL tumor suppressor gene, leading to constitutive stabilization of multiple hypoxia-inducible factors (HIFs). Notably, HIF-2α plays a prominent role in clear-cell renal cell carcinoma (ccRCC) tumorigenesis [1].

As a consequence of this molecular dysregulation, patients with VHL syndrome develop a spectrum of benign and malignant neoplasms, including bilateral ccRCC, central nervous system hemangioblastomas, pancreatic neuroendocrine tumors, and pheochromocytomas. Tumors often arise early in life, recur over time, and involve multiple organs, resulting in a lifelong reliance on repeated surgical interventions and substantial cumulative morbidity [2,3].

Beyond hereditary disease, loss of VHL function is also a defining molecular feature of sporadic ccRCC, with somatic VHL inactivation reported in approximately 70-90% of cases [3]. Renal cell carcinoma (RCC) accounts for the majority of primary kidney malignancies and represents one of the most commonly diagnosed solid tumors worldwide [2]. Reported rates of RCC have increased over recent decades, reflecting both enhanced detection through imaging and broader demographic and metabolic trends. According to GLOBOCAN 2022, RCC accounts for approximately 435,000 new cases and 15600 deaths annually, underscoring its substantial contribution to cancer-related morbidity and mortality [2].

Belzutifan represents a targeted therapeutic approach that directly addresses the shared molecular pathology linking VHL syndrome and sporadic ccRCC. By selectively inhibiting HIF-2α, belzutifan targets a key transcriptional driver of disease biology, offering a mechanistically distinct alternative to traditional VEGF-directed therapies [3]. Historically, systemic treatments have played a limited role in VHL due to modest efficacy, cumulative toxicity, and failure to modify the underlying disease process. The clinical relevance of HIF-2α inhibition was demonstrated in the phase II LITESPARK-004 trial, in which oral belzutifan achieved objective responses and durable tumor control in patients with VHL-associated RCC, leading to FDA approval in 2021 and establishing HIF-2α inhibition as a transformative therapeutic paradigm in VHL disease [4-6].

Methods

A structured literature search was conducted to identify studies evaluating belzutifan in VHL-associated tumors. Searches were performed in PubMed/MEDLINE, Embase, ClinicalTrials.gov, and the U.S. Food and Drug Administration (FDA) database from database inception through December 2025. Search terms included “belzutifan,” “HIF-2α inhibitor,” “renal cell carcinoma,” “Von Hippel-Lindau disease,” “VHL-associated tumors,” “hypoxia-inducible factor,” "adverse effects", and “LITESPARK trials,” used individually or in combination with Boolean operators (AND, OR).

To ensure completeness, backward citation searching was performed by reviewing the reference lists of selected articles.

Studies were included if they evaluated the mechanism, efficacy, or safety of belzutifan in VHL-associated tumors and consisted of clinical trials, observational studies, or relevant preclinical data. Review articles and regulatory documents were also included to provide clinical context. Studies were excluded if they were non-English, not relevant to belzutifan or the HIF-2α pathway, or represented duplicate or overlapping datasets. Due to heterogeneity in study design and reported outcomes, findings were synthesized qualitatively. Clinical trial data were obtained from ClinicalTrials.gov, and regulatory information was obtained from the United States Food and Drug Administration (FDA) database.

## Review

Pathophysiology of the VHL gene

The VHL gene encodes a tumor suppressor protein that plays a critical role in regulating cellular responses to oxygen availability. Rather than acting directly on cell-cycle control, the VHL protein functions as a molecular regulator of hypoxia-responsive signaling, ensuring that these pathways remain inactive under normal physiological conditions. Disruption of this regulatory function underlies the development of hereditary VHL syndrome and contributes to tumorigenesis across multiple organ systems [7].

The interaction between VHL and HIFs constitutes a central mechanism of cellular oxygen sensing. When VHL activity is preserved, HIF-α protein levels are tightly controlled, thereby restricting hypoxia-responsive gene expression to settings of genuine oxygen deprivation. This regulation is essential for maintaining balanced angiogenesis, metabolic homeostasis, and controlled cellular survival signaling. Loss of this regulatory axis results in sustained hypoxia signaling that drives pathological processes, including tumor development and chronic kidney disease [7,8].

In the absence of functional VHL, hypoxia signaling becomes uncoupled from ambient oxygen levels, leading to a state commonly referred to as pseudohypoxia. Despite normoxic conditions, HIF-driven transcription remains persistently active, resulting in increased expression of genes that promote angiogenesis, metabolic adaptation, and resistance to apoptosis. This aberrant signaling environment creates a permissive biological context for tumor initiation, growth, and progression in VHL-associated neoplasms [8,9].

Persistent activation of hypoxia-responsive pathways also induces metabolic reprogramming that favors glycolytic energy production over mitochondrial oxidative phosphorylation. This shift supports ATP generation under conditions of inefficient oxygen utilization and provides a survival advantage to tumor cells. These metabolic adaptations contribute to sustained proliferation and tumor progression [8].

Among the HIF family members, HIF-2α has emerged as a dominant oncogenic driver in VHL-deficient states. Unlike HIF-1α, which primarily mediates acute hypoxic responses, HIF-2α is more closely associated with chronic hypoxia adaptation and prolonged transcriptional activation. Its selective activation contributes to sustained angiogenic signaling, metabolic flexibility, and tumor maintenance, positioning HIF-2α as a central driver of disease biology in VHL-deficient states [8].

The clinical relevance of these molecular events is underscored by the observation that biallelic VHL inactivation is present in the vast majority of sporadic ccRCCs [9,10]. This shared biological foundation explains the highly vascular nature of these tumors and provides a unifying mechanistic link between hereditary VHL disease and sporadic renal malignancy. Consequently, dysregulation of the VHL-HIF pathway represents not only a defining feature of disease pathogenesis but also an important therapeutic target in RCC and related hypoxia-driven tumors [7,8].

Current clinical management of VHL-associated manifestations

*Renal Manifestations*

In VHL disease, renal lesions are characteristically bilateral and multifocal, with numerous microscopic tumor foci often present. Nephron-sparing surgery is usually considered when the largest lesion reaches more than approximately 3 cm in diameter, as the risk of metastatic progression remains low below this threshold. Nevertheless, early surgical intervention may be considered for smaller tumors demonstrating rapid growth. Given the multiplicity of renal lesions, a single operative procedure may involve the excision of dozens of tumors, and repeat surgeries at intervals of three to five years are frequently required [11].

The therapeutic landscape of advanced ccRCC has evolved substantially with the introduction of antiangiogenic agents and immunotherapies. In particular, immune checkpoint inhibitors (ICIs) targeting programmed death 1 (PD-1) or programmed death ligand 1 (PD-L1), administered either in combination with cytotoxic T-lymphocyte-associated protein 4 (CTLA-4) inhibitors or with vascular endothelial growth factor tyrosine kinase inhibitors (VEGF-TKIs), have become central components of first-line treatment for advanced ccRCC. These combination strategies have demonstrated significant clinical benefit in multiple phase III trials and have contributed to a shift in standard-of-care therapy from single-agent VEGF-TKIs to ICI-based combination regimens.

Despite these advances, advanced ccRCC remains a challenging disease to manage, as the majority of patients ultimately experience disease progression and require additional systemic therapy [12].

CNS Manifestations

VHL treatment relies on active surveillance protocols for early detection and monitoring of the disease. Hemangioblastomas can occur in both the brain and spine. These lesions are monitored, and when neurologic symptoms develop, a surgical approach is favored. According to a prospective natural history observational study of VHL patients with CNS hemangioblastomas, these tumors demonstrated a saltatory growth pattern. The unpredictable growth behavior of hemangioblastomas poses a challenge in determining surgical indications. Tumor size may remain stable for prolonged periods, followed by unpredictable growth. Symptom development remains the primary indication for surgical intervention. For asymptomatic patients, follow-up with an MRI is indicated. Some authors also consider growing tumors without neurologic symptoms to be an indication for surgical resection [13].

Retinal Manifestations

According to the American Academy of Ophthalmology (AAO) VHL guidelines, patients who test positive for VHL and high-risk patients with a positive family history are candidates for dilated eye examinations. Screening starts before 1 year of age and is performed every 6-12 months. Once the patient reaches 30 years of age, annual screening is recommended. Traditional treatments for RH include laser photocoagulation, cryotherapy, photodynamic therapy, radiotherapy, pars plana vitrectomy with local resection, and adjuvant anti-VEGF therapy with or without corticosteroid injections [14]. Since VHL encompasses a wide range of diseases requiring lifelong follow-up and repeated surgical interventions, there is a need for systemic therapies that control VHL-related neoplasms while minimizing surgical procedures, preserving organ function, and improving quality of life (QoL) in patients [11].

Mechanism of action

HIF-2α Inhibition by Belzutifan

Cellular regulation of HIF-2α is governed by oxygen-dependent post-translational modification. Under normoxic conditions, prolyl hydroxylase domain enzymes (PHDs) catalyze hydroxylation of specific proline residues within HIF-2α, generating a recognition motif for the VHL protein (pVHL). Acting as the substrate-recognition component of an E3 ubiquitin ligase complex, pVHL facilitates polyubiquitination of hydroxylated HIF-2α and targets it for rapid proteasomal degradation, thereby maintaining low basal HIF-2α activity [15].

In contrast, reduced oxygen availability prevents prolyl hydroxylation, rendering pVHL unable to engage HIF-2α and resulting in stabilization of the transcription factor. Accumulated HIF-2α subsequently heterodimerizes with its constitutively expressed binding partner HIF-β, also known as aryl hydrocarbon receptor nuclear translocator (ARNT), forming an active transcriptional complex. This complex induces expression of hypoxia-responsive genes, including angiogenic mediators such as VEGF and platelet-derived growth factor (PDGF), as well as metabolic regulators that enhance glucose uptake and glycolysis through transporters such as GLUT1 and GLUT3 [15]. 

When the VHL function is lost, hypoxia-inducible signaling persists despite normal oxygen tension, creating a pathological transcriptional state commonly described as cellular pseudohypoxia. This sustained HIF-2α-driven program promotes angiogenesis, metabolic reprogramming, and tumor invasion, thereby supporting oncogenic progression. In this context, HIF-2α functions as a central mediator of tumor-sustaining hypoxia signaling.

Among the downstream effectors of HIF-2α activation, VEGF plays a dominant role in mediating tumor-associated angiogenesis. Pathologic overexpression of VEGF stimulates endothelial cell proliferation, migration, and survival, enhances vascular permeability, and contributes to the formation of an aberrant tumor vasculature that supports continued tumor growth and dissemination [16].

Belzutifan is a selective HIF-2α inhibitor that works by disrupting the formation of the HIF transcriptional complex by impairing the formation of the active hypoxia-inducible transcriptional complex of HIF-2α and HIF-β. Rather than reducing HIF-2α expression, the drug interferes with its functional activation, thereby suppressing downstream transcriptional programs that promote angiogenesis, erythropoiesis, metabolic adaptation, and tumor survival [3]. This targeted inhibition directly addresses the pseudohypoxic signaling state that characterizes VHL-deficient tumors. 

The therapeutic relevance of HIF-2α inhibition is further supported by evidence demonstrating that HIF-2α plays a more prominent role than HIF-1α in chronic hypoxia adaptation and tumorigenesis in ccRCC. Whereas HIF-1α primarily mediates acute hypoxic responses, HIF-2α is more closely associated with sustained oncogenic signaling, long-term tumor survival, and disease progression. Selective inhibition of HIF-2α therefore represents a rational and disease-specific therapeutic strategy in both hereditary VHL disease and sporadic ccRCC [8,15].

**Figure 1:**
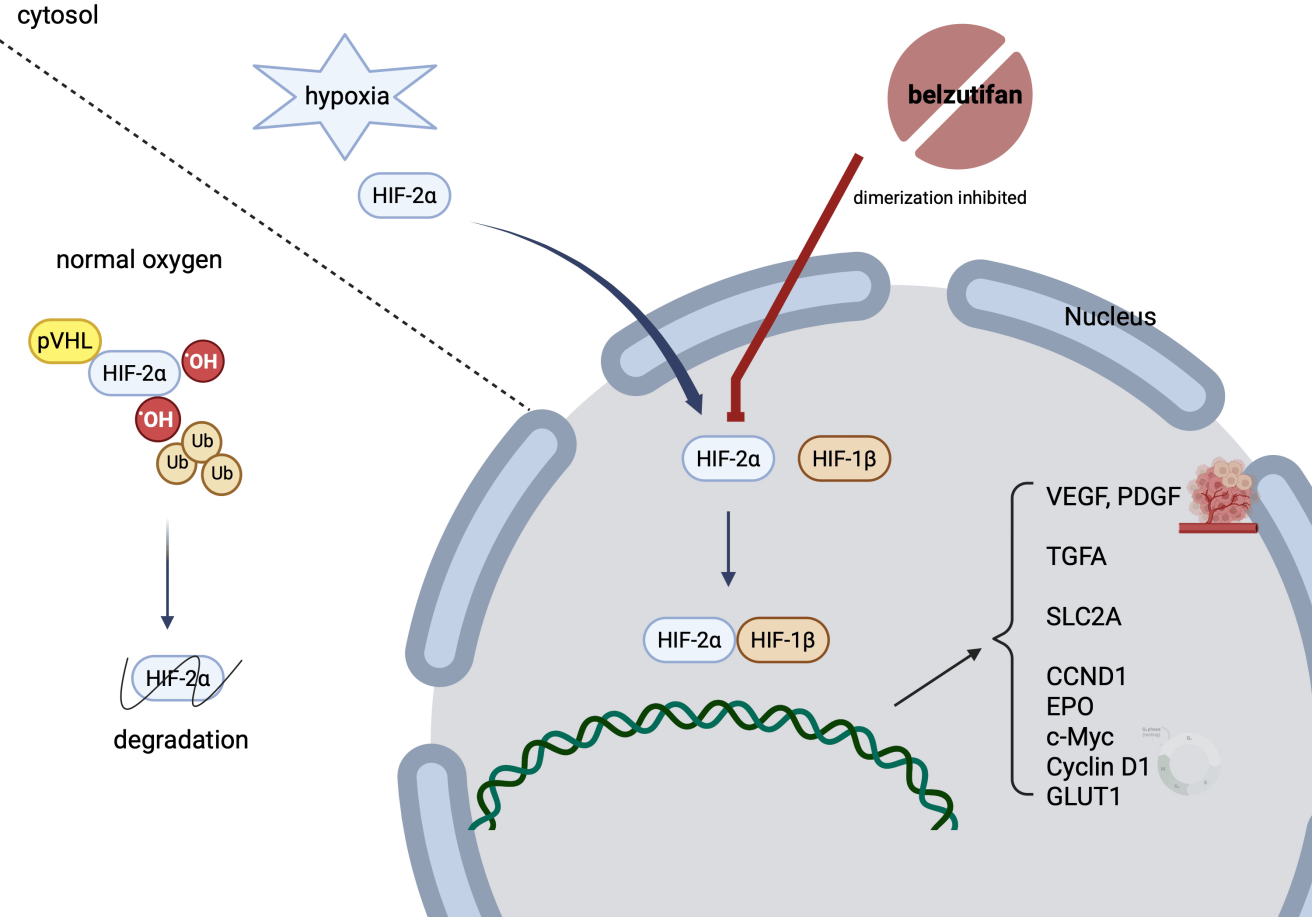
Regulation of the VHL/HIF pathway under normoxic and hypoxic conditions and mechanism of HIF-2α inhibition by belzutifan. Figure created by the authors using BioRender.com (BioRender Inc., Toronto, ON, Canada). This is an original figure and has not been reproduced from any external source. VHL, von Hippel-Lindau; HIF, hypoxia-inducible factor

The molecular regulation of the VHL/HIF pathway under normoxic and hypoxic conditions, as well as the mechanism of HIF-2α inhibition by belzutifan, are summarized in Figure 1.

Clinical pharmacology of belzutifan

*Pharmacodynamic Profile*

At the molecular level, belzutifan binds with high affinity to a specific hydrophobic pocket (PAS-B domain) of the HIF-2α subunit. Preclinical studies have demonstrated dissociation constants (Kd) in the low nanomolar range (~20-30 nM), indicating strong target binding. This binding allosterically destabilizes the heterodimerization of HIF‑2α with its obligate partner, the ARNT (also known as HIF‑1β), thereby disrupting the formation of the active transcription factor complex. Biochemical assays demonstrate that HIF‑2α transcriptional activity is inhibited with comparable potency(IC50 ~20-30 nM), resulting in reduced expression of genes that respond to hypoxia. These findings support potent direct target engagement by belzutifan at clinically relevant concentrations [17].

Clinical studies show that belzutifan produces reproducible pharmacodynamic changes consistent with inhibition of HIF-2α signaling. In a phase 1/2 first-in-human study, reductions in circulating erythropoietin (EPO) levels were observed shortly after treatment initiation and served as a measurable indicator of on-target biological activity. Reduction of EPO level reaches a plateau at clinically relevant doses, particularly at the approved dose of 120 mg once daily, indicating sustained HIF-2α inhibition without additional biological benefit at higher exposures. Decreases in hemoglobin occur more gradually during continued treatment and subsequently reach a stable plateau, reflecting sustained suppression of EPO signaling. These effects appear to reach maximal biological impact at the approved dose, supporting the selected dosing regimen. Pharmacodynamic markers are not used to assess treatment response, which remains radiologically determined, but they confirm on-target biological activity during belzutifan therapy [18].

Pharmacokinetic Profile

Population pharmacokinetic analyses from phase I and II clinical studies demonstrate that belzutifan follows a linear two-compartment model with first-order absorption and elimination, allowing predictable systemic concentrations across the evaluated dosing range. The estimated apparent clearance is approximately 7.3 L/hour, with an apparent volume of distribution of approximately 130 L and a terminal elimination half-life of ~12.4 hours, supporting once-daily dosing. Steady-state concentrations are achieved within several days of continuous administration [19].

Belzutifan exposure is not meaningfully influenced by age, sex, race, body weight, or mild to moderate renal or mild hepatic impairment, indicating that routine dose adjustments are generally not required for these patient groups. Although food intake and the final marketed formulation significantly reduce the absorption rate constant, neither has a clinically meaningful effect on overall exposure, as demonstrated by the area under the curve (AUC), at steady state. Simulation analyses suggest that occasional delayed or missed doses have a limited impact on steady-state average exposure, supporting flexible dosing instructions without routine dose compensation. Despite fixed dosing, belzutifan exhibits moderate interindividual variability in systemic exposure, with an AUC coefficient of variation of approximately 50%, which is not explained by demographic or organ function differences [19].

In contrast, individuals with reduced metabolic activity of UGT2B17 and CYP2C19, the principal enzymes responsible for belzutifan clearance, may exhibit increased systemic exposure. These findings support a clinical approach focused on careful safety monitoring rather than routine genotype-guided dose adjustment [19].

Dose selection and optimization of belzutifan

Dose selection for belzutifan was established through a stepwise integration of tolerability, pharmacodynamic target engagement, and early efficacy signals in a first-in-human phase I study. While escalation to higher daily doses did not identify a formal maximum tolerated dose, exposure-response evaluation indicated limited incremental pharmacokinetic or pharmacodynamic benefit at dose levels above 120 mg once daily. Reductions in EPO levels, reflecting downstream inhibition of HIF-2α signaling, were evident across evaluated dose ranges and appeared to stabilize at doses of 120 mg or higher, suggesting attainment of maximal biological effect without added benefit from further dose escalation. Dose levels exceeding this threshold were associated with increased frequency of mechanism-related adverse effects, most notably anemia and hypoxia, while failing to produce meaningful gains in antitumor efficacy [18].

Support for this dosing strategy was subsequently provided by phase II clinical evaluation in patients with VHL-associated tumors, in which continuous treatment with belzutifan at 120 mg once daily resulted in sustained clinical responses across VHL-associated RCC, VHL-associated CNS hemangioblastomas, and VHL-associated pancreatic neuroendocrine tumors [9]. Importantly, long-term treatment confirmed that mechanism-related toxicities were manageable with supportive measures and temporary treatment interruption, rather than dose escalation or discontinuation. Collectively, these observations support 120 mg once-daily dosing as an effective balance between durable HIF-2α inhibition and acceptable long-term tolerability, forming the basis for current clinical dosing recommendations [9].

Clinical indications for belzutifan

VHL-Associated tumors 

Belzutifan received FDA approval for the treatment of adult patients with VHL disease who do not require immediate surgery for VHL-associated RCC, CNS hemangioblastomas, or pancreatic neuroendocrine tumors. This indication reflects its role as a systemic therapy intended to modify disease progression rather than replace established surgical management. As a HIF-2α inhibitor, belzutifan targets the dysregulated VHL-HIF signaling pathway, consistent with a mechanistic rationale for its clinical use. Given the need for repeated surgical interventions over the lifetime of many patients with VHL disease, this drug serves as a systemic therapy directed toward stabilizing tumor growth and thus potentially reducing the frequency or preventing surgical intervention altogether [20,21].

*Advanced ccRCC After Prior Immunotherapy​​*​​​​​*/TKI Therapy *

Belzutifan is also considered for use in adults with ccRCC who have experienced disease progression following prior treatment with immune checkpoint inhibitors and VEGF-targeted therapies, positioning it for use beyond the first-line setting [22,23]. While the regulatory label does not formally designate belzutifan as a second-line agent, its approved indication supports use in later lines of therapy following failure of prior systemic treatments. The approved indication also encompasses patients with unresectable, locally advanced, or metastatic ccRCC who have progressed following systemic therapy or cytoreductive nephrectomy, supported by efficacy data from the phase III LITESPARK-005 trial demonstrating improved outcomes with belzutifan [22,23].

*Absence of an Approved Adjuvant Role*

Although belzutifan's potential role in the adjuvant setting is being evaluated in ongoing clinical trials, including combination strategies with pembrolizumab, definitive evidence and regulatory approval are lacking. Importantly, prior surgical resection is not required for belzutifan's use within approved indications, and treatment may be offered in unresectable or metastatic disease irrespective of nephrectomy status [3].

Pharmacogenetic Considerations

Beyond disease indication and treatment settings, variability in belzutifan exposure across patients has important implications for toxicity monitoring in clinical practice. Population-based pharmacokinetic modeling analysis indicates that variability in belzutifan exposure is largely independent of demographic or clinical characteristics but is strongly influenced by genetic differences in drug metabolism. In particular, patients who are poor metabolizers for both UGT2B17 and CYP2C19 exhibit substantially increased systemic exposure, with estimated belzutifan AUC values approximately 3.2-fold higher than those observed in non-poor metabolizers [19].

The clinical relevance of elevated belzutifan exposure is underscored by its mechanism of action, which is associated with predictable on-target toxicities such as anemia and hypoxia. Consequently, the current guidelines emphasize close clinical monitoring for toxicity, while routine pharmacogenetic screening before the initiation of treatment is not advised. Existing recommendations prioritize standard dosing with vigilant toxicity surveillance, with clinical management driven by observed adverse effects rather than genetic status alone [4,15].

Clinical use of belzutifan across VHL-associated tumors

VHL-Associated RCC

Among the multisystem manifestations of VHL disease, RCC represents the greatest long-term clinical challenge because of its cumulative surgical burden and risk of metastatic progression. Individuals with VHL disease have an estimated 70% lifetime risk of developing RCC, which is typically of the clear cell subtype, and tumors exceeding 3 cm in diameter are associated with an increased risk of metastasis. In contrast to sporadic RCC, renal tumors in VHL disease arise bilaterally and recur over time, frequently necessitating repeated surgical or ablative interventions and contributing to progressive renal dysfunction, chronic kidney disease, and, in some cases, dialysis [16].

Despite intensive surveillance and intervention, metastatic RCC remains a leading cause of mortality in this population. Concurrent involvement of the central nervous system (CNS) and retina further contributes to cumulative organ morbidity, including neurological impairment, vision loss, and reduced QoL. Accordingly, optimal therapeutic goals in VHL disease include durable tumor cytoreduction, suppression of new lesion formation, and prevention of disease progression, to minimize repeated invasive procedures and long-term complications such as renal failure or vision loss [16].

The pivotal LITESPARK-004 trial established the clinical role of belzutifan in this context. This phase II study enrolled 61 patients with localized VHL-associated RCC, who received belzutifan 120 mg orally once daily. The trial demonstrated significant clinical activity against RCC as well as several non-RCC VHL-associated tumors, forming the basis for regulatory approval. Among patients with VHL-associated RCC, 49% achieved a confirmed objective response, with most demonstrating measurable tumor shrinkage, confirming the antitumor efficacy of belzutifan [16].

Although the precise mechanisms of resistance to belzutifan have not been fully elucidated, prior preclinical studies and early-phase clinical trials of earlier HIF-2α inhibitors, such as PT2399 and PT2385, suggest that acquired resistance may arise through a HIF-2α G323E gatekeeper mutation, which interferes with HIF-2α dimer dissociation [9].

Extended follow-up data from the LITESPARK-004 trial further support the durability of response. Between May 31, 2018, and March 29, 2019, 61 patients were enrolled, and 36 patients (59%) remained on belzutifan therapy as of April 3, 2023. The median duration of follow-up was 49.9 months (IQR, 48.9-52.2). An objective response in RCC was observed in 41 of 61 patients (67%; 95% CI, 54-79), including complete responses in seven patients (11%) and partial responses in 34 patients (56%). Grade 3 treatment-related adverse events occurred in 11 patients (18%), most commonly anemia (11%), followed by fatigue (5%), urinary tract infection (2%), hypoxia (2%), and blistering (2%). No grade 4 or 5 treatment-related adverse events were reported. Serious treatment-related adverse events occurred in four patients (7%), consisting of anemia, urinary tract infection, intracranial hemorrhage, and hypoxia. These long-term data demonstrate durable tumor control with increasing depth of response over time and a consistently favorable safety profile, supporting belzutifan as a long-term disease-modifying therapy in VHL-associated RCC [5].

CNS Hemangioblastomas

The availability of effective systemic therapy may lessen reliance on repeated surgical procedures, as extra-renal manifestations of VHL disease represent a significant source of long-term morbidity and mortality. In the LITESPARK-004 trial, 30% of patients with CNS hemangioblastomas and 91% of patients with pancreatic neuroendocrine tumors achieved objective responses, indicating meaningful antitumor activity of belzutifan across multiple VHL-associated tumor types [16].

Reductions in lesion size are expected to translate into fewer indications for surgical intervention, as tumor size is a critical determinant of operative decision-making. Notably, many patients enrolled in LITESPARK-004 had undergone multiple surgical or ablative procedures before initiating belzutifan therapy, whereas only three patients required tumor-directed interventions after treatment initiation. These findings formed the foundation for regulatory approval of belzutifan, even though its optimal positioning within the VHL treatment paradigm continues to evolve. Overall, the trial demonstrated that belzutifan may delay or even obviate the need for repeated surgeries, which are associated with substantial cumulative morbidity [9].

A dedicated CNS hemangioblastoma follow-up analysis from LITESPARK-004 included 50 of 67 screened patients (82%) with at least one measurable CNS hemangioblastoma at baseline, encompassing a total of 184 lesions. The median follow-up duration was 38.8 months (IQR, 36.7-40.1). Using two predefined assessment approaches, objective responses were observed in 44% (95% CI, 30-59) and 76% (95% CI, 55-91) of evaluable patients, respectively. Grade 3-5 all-cause adverse events occurred in 46% of patients, with anemia being the most common grade 3 event (12%). Grade 4 events occurred in two patients (4%), including retinal vein occlusion and embolism. Two deaths were reported due to adverse events not considered treatment-related (suicide and toxicity from multiple agents) [24].

Additional single-center retrospective studies further support these findings. In one series of seven patients with 25 CNS hemangioblastomas, treated with belzutifan 120 mg daily, an objective response rate of 71% was observed, with partial responses in 71.4% of patients and stable disease in 28.5%, and no complete responses were reported [25]. Another single-center experience reported a median treatment duration of 11 months, with all patients demonstrating radiographic responses; the median time to initial response was 3 months, and the median time to maximal response was 8 months [26]. Collectively, these studies consistently demonstrate significant and sustained antitumor activity of belzutifan in CNS hemangioblastomas [24-26].

Retinal Hemangioblastomas

Retinal hemangioblastomas (RHs) in VHL disease are a major cause of visual impairment and ocular morbidity, particularly when lesions are advanced or involve critical structures such as the optic nerve or macula. Historically, management has relied on locally destructive therapies, including laser photocoagulation, cryotherapy, photodynamic therapy, radiotherapy, pars plana vitrectomy with tumor resection, and adjunctive intravitreal anti-VEGF or corticosteroid injections. These approaches are frequently associated with significant visual morbidity, especially for posteriorly located lesions [14].

At the University of Iowa, seven patients (12 eyes) with VHL-associated RHs treated with belzutifan were identified. Six patients received an initial dose of 120 mg once daily, while one pediatric patient was initiated at 80 mg daily. A parallel literature review identified seven published studies describing ocular outcomes in a total of 21 patients with VHL-associated RHs. The mean reported age was 36 years (range, 11-71 years), with available gender data indicating six male and three female patients. Primary indications for belzutifan therapy included RCC (n = 13), RHs (n = 4), CNS hemangioblastomas (n = 4), and pancreatic neuroendocrine tumors (n = 1).

All patients initially received belzutifan at 120 mg daily, with subsequent dose reductions to 80 mg in three patients and 40 mg in one patient. Anemia developed in five patients during treatment. Importantly, all ocular tumors demonstrated a size reduction, indicating consistent radiographic response. Follow-up durations ranged from 1 to 30 months.

Given the limited but encouraging data, individualized surveillance is currently recommended for patients receiving belzutifan for RHs. Patients with large or vision-threatening tumors may require monthly monitoring, particularly during dose interruptions or reductions, whereas those with small, peripheral lesions may be followed at longer intervals. In early RCC approval studies, imaging was performed every three months, and this interval has been adopted for ocular monitoring in current clinical practice [14].

Overall, belzultifan use in VHL, especially in RCC, CNS hemangioblastoma, and retinoblastoma, showed both consistently in tumor reduction and the possibility of long-term treatment with minimal serious adverse events.

Clinical development and regulatory approval of belzutifan in RCC

FDA Approval

Improved understanding of dysregulation within the VHL-HIF signaling pathway has provided the biological rationale for belzutifan as an effective therapeutic option in both hereditary and sporadic forms of RCC. Globally, RCC contributes to approximately 134,000 deaths each year, including nearly 14,000 in the United States. The clear cell variant constitutes roughly three-quarters of all diagnoses and is the most extensively studied in current therapeutic research [3].

In 2021, belzutifan became the first approved pharmacologic inhibitor of HIF-2α following FDA authorization for use in adults with VHL disease-associated tumors, including RCC, CNS hemangioblastomas, and pancreatic neuroendocrine tumors, when immediate surgical intervention was not indicated. The FDA subsequently expanded the approved indication for belzutifan in 2023 to include adults with advanced RCC whose disease had progressed after prior systemic therapy. This regulatory decision was informed by results from the phase III LITESPARK-005 trial, which evaluated belzutifan in comparison with everolimus in patients with previously treated advanced ccRCC. The trial results indicated superior progression-free survival and objective response rates with belzutifan [27].

Current first-line management of metastatic RCC typically relies on immune checkpoint inhibitors targeting the PD-1/PD-L1 axis, often in combination with VEGF-directed tyrosine kinase inhibitors, which together form the foundation of systemic therapy across treatment settings. Approval of belzutifan for previously treated advanced RCC introduces a therapeutic option with a novel mechanism of action and a safety profile that differs from existing agents, allowing for greater individualization of treatment selection [28].

Phase I LITESPARK-001 in Pretreated ccRCC

The efficacy of belzutifan was first evaluated in LITESPARK-001, an open-label phase I trial employing a traditional 3 + 3 dose escalation design followed by an expansion phase. The study enrolled patients with previously treated advanced ccRCC across seven centers. Participants received belzutifan 120 mg orally once daily and were treated until disease progression, unacceptable toxicity, or voluntary withdrawal.

Among the 55 treated patients, 25% achieved an objective response, with several responses lasting longer than 40 months. Treatment-related adverse events occurred in 53 patients (96%), with grade 3 toxicities reported in 22 patients (40%). The most frequent grade 3 adverse events were anemia (24%) and hypoxia (13%). Importantly, no grade 4 or 5 treatment-related adverse events were observed. These findings supported belzutifan’s durable antitumor activity and manageable safety profile in patients with heavily pretreated ccRCC [29].

Phase III LITESPARK-005 in Pretreated ccRCC

LITESPARK-005 was a randomized, open-label, multicenter phase III study evaluating belzutifan in patients with advanced ccRCC who had previously been treated with both immune checkpoint inhibitors and VEGF-targeted therapies. The trial included 374 patients receiving belzutifan and 372 patients receiving everolimus. Patients were randomized to receive either belzutifan 120 mg or everolimus 10 mg orally once daily, continuing therapy until disease progression or the development of intolerable toxicity appeared.

At the initial interim analysis (median follow-up of 18.4 months), median progression-free survival was 5.6 months in both treatment groups. However, belzutifan demonstrated a remarkably higher objective response rate compared with everolimus (21.9% vs. 3.5%; *P* < 0.001), indicating superior tumor shrinkage. At the second interim analysis (median follow-up of 25.7 months), overall survival was similar between the two groups (21.4 months with belzutifan vs. 18.4 months with everolimus), and the difference did not meet prespecified thresholds for statistical significance. Grade > 3 treatment-related adverse events occurred at comparable rates (61.8% with belzutifan vs. 62.5% with everolimus), although treatment discontinuation due to toxicity was less frequent in the belzutifan group (5.9% vs. 14.7%).

Collectively, these findings demonstrate a significantly higher objective response rate with belzutifan compared with everolimus in a heavily pretreated population, while median PFS and OS were similar at interim analyses (5.6 months). Belzutifan was generally well tolerated with a manageable safety profile, supporting its role as a treatment option in this setting [23].

Current Position of Belzutifan in the Treatment of ccRCC

With FDA approval, belzutifan has been incorporated into evolving treatment algorithms for RCC as therapeutic strategies expand beyond VEGF tyrosine kinase inhibitors and immune checkpoint inhibitors. Current clinical evidence supports its use in the second-line setting and beyond, particularly in patients with advanced RCC who have progressed after frontline ICI/TKI combination regimens. This positioning is supported by data from the LITESPARK-005 trial. Belzutifan offers a mechanistically distinct option, especially for patients who experience intolerance or cumulative toxicities related to VEGF-TKIs, making it a meaningful addition to contemporary RCC management [3,23].

Emerging investigational uses of belzutifan

Neuroendocrine and Neural Crest-Derived Tumors

Although clinical data supporting belzutifan use beyond RCC are currently limited, growing interest has emerged due to the dependence of several tumor types on dysregulated VHL-HIF signaling. Belzutifan is currently under evaluation in phase II clinical studies involving advanced or metastatic pheochromocytoma, paraganglioma, and pancreatic neuroendocrine tumors, malignancies that are often characterized by alterations in cellular oxygen-sensing pathways. However, the precise contribution of HIF-2α as a primary oncogenic driver in these disease contexts has not yet been clearly defined [30].

Gastrointestinal and Hepatobiliary Malignancies

The LITESPARK-016 study is investigating belzutifan administered alone or in combination with pembrolizumab and lenvatinib across several advanced solid malignancies, including hepatocellular carcinoma, colorectal cancer, pancreatic ductal adenocarcinoma, and biliary tract tumors. Hypoxic signaling within the tumor microenvironment has been implicated in prostate cancer progression and resistance to therapy, with activation of HIF pathways contributing to these processes [31].

Prostate Cancer

Belzutifan is also being explored in prostate cancer. One ongoing study evaluates its use in the neoadjuvant setting alongside androgen-deprivation therapy and abiraterone, while another examines its role in metastatic castration-resistant prostate cancer. Results from these trials are pending, with expected reporting timelines extending to 2027.

Hypoxic signaling within the tumor microenvironment has been implicated in prostate cancer progression and resistance to therapy, with activation of HIF pathways contributing to these processes. HIF-2α promotes transcription of genes involved in angiogenesis, metabolic adaptation, cell survival, and androgen-independent growth, and its increased activity has been associated with aggressive and treatment-refractory disease. Preclinical studies demonstrate that HIF-2α inhibition reduces tumor growth and angiogenesis in hypoxic prostate cancer models. These findings provide a mechanistic rationale for evaluating belzutifan in HIF-driven prostate cancer, particularly in advanced or castration-resistant settings, where hypoxia contributes to disease progression and resistance to androgen receptor-targeted therapies [32].

Nonmalignant and Rare HIF-Driven Disorder

In addition to oncologic indications, inhibition of HIF-2α may hold therapeutic potential in select non-malignant disorders involving dysregulated oxygen-sensing mechanisms. These include hereditary erythrocytosis and pulmonary hypertension, where HIF-mediated signaling has shown pathogenic relevance in preclinical studies. In addition, belzutifan has been successfully used in an adolescent with Pacak-Zhuang syndrome, a rare disorder caused by activating mutations in EPAS1, highlighting its potential role in select HIF-driven genetic conditions.

Pacak-Zhuang syndrome is a rare hereditary disorder caused by gain-of-function mutations in EPAS1, resulting in constitutive activation of HIF-2α despite normal oxygen tension. This persistent pseudohypoxic signaling drives excess EPO production, leading to polycythemia, and promotes the development of paragangliomas, pheochromocytomas, and somatostatinomas. Because tumorigenesis in this syndrome is directly dependent on aberrant HIF-2α signaling, targeted inhibition represents a rational therapeutic approach. Belzutifan, a selective HIF-2α inhibitor, blocks heterodimerization of HIF-2α with HIF-1β, thereby suppressing transcription of hypoxia-responsive genes involved in angiogenesis, metabolism, and erythropoiesis. Clinical reports have demonstrated reductions in hemoglobin levels and tumor stabilization in EPAS1-mutated disease, supporting belzutifan as a precision therapy that targets the molecular driver of Pacak-Zhuang syndrome [33].

*Pediatric Considerations*

There is currently no consensus regarding the use of belzutifan in pediatric patients. Although RH often manifests early in life among individuals with VHL disease, therapeutic options in children remain limited, and evidence guiding age-specific dosing, long-term safety, and clinical durability of belzutifan is scarce [34].

Available evidence regarding pediatric use of belzutifan is derived from a small number of case series reporting favorable clinical and radiographic outcomes. Across five reported pediatric cases, belzutifan demonstrated activity against retinal and CNS hemangioblastomas at daily doses ranging from 60 to 120 mg [34].

In an 11-year-old female with partial VHL gene deletion and concurrent retinal and cerebellar hemangioblastomas, treatment resulted in improved visual acuity, regression of feeder vessels, reduction in hemangioblastoma size, and improvement in macular peripheral changes. A similar multisystem response was observed in a 17-year-old male with retinal and intracranial hemangioblastomas treated with belzutifan 120 mg daily, who experienced resolution of macular edema and reduction in the size of the three largest intracranial lesions on magnetic resonance imaging after 18 months of therapy [34].

Responses were also documented at lower doses. A 10-year-old male with retinal hemangioblastoma treated with belzutifan 80 mg daily demonstrated tumor regression and decreased fibrosis after 16 months. In a 16-year-old female with orbital and intracranial hemangioblastomas as well as pancreatic cysts, treatment with belzutifan at doses ranging from 80 to 120 mg daily led to a reduction in cerebellar lesion size from 14 × 14 × 17 mm to 8 × 8 × 11 mm after 10 months. Similarly, a 16-year-old male with retinal and intracranial hemangioblastomas treated with 60-80 mg daily showed reductions in cervicomedullary junction and cerebellar lesions from 14 to 10 mm and from 10 to 8 mm, respectively [34].

These limited case reports suggest that belzutifan is generally well tolerated in pediatric patients; however, available data remain insufficient to draw definitive conclusions regarding safety or efficacy in this population. Although these findings suggest that belzutifan may represent a promising systemic therapeutic option for retinal and CNS tumors associated with VHL disease in pediatric patients, the current evidence remains limited to small observational reports. Larger prospective studies are therefore required to define optimal dosing, characterize long-term safety, and establish sustained efficacy in this population [34].

Taken together, a broad range of clinical studies has evaluated belzutifan across multiple disease settings, including RCC, pheochromocytoma, paraganglioma, and other HIF-2α-related tumors [35-39]. Population pharmacokinetic and exposure-response analyses have established the optimal 120-mg once-daily dose and clarified predictors of hypoxia-related toxicity, supporting its clinical applicability [35,37]. Concurrently, dose-escalation and safety-expansion studies have demonstrated the drug’s broader therapeutic potential across tumor types [37-39]. Patient-reported outcome (PRO) data from randomized trials further indicate stable symptoms and preserved QoL during therapy [36]. These findings provide a framework for understanding belzutifan’s clinical indications and ongoing development, with completed RCC trials summarized in Table 1 [4,9,23,27,29,35-39], and active trials across various tumors are outlined in Tables 2-3.

**Table 1:** Completed (active, not recruiting) or reported clinical trials of belzutifan. Data compiled by authors from published clinical trials and regulatory sources [4,9,23,27,29,35-39]. AE, adverse events; BID, twice daily; ccRCC, clear cell renal cell carcinoma; CI, confidence interval; CNS, central nervous system; CR, complete response; HR, hazard ratio; ICI, immune checkpoint inhibitor; MDR, median duration of response; MFU, median follow-up; MPFS, median progression-free survival; ORR, objective response rate; OS, overall survival; PD, progressive disease; PFS, progression-free survival; pNET, pancreatic neuroendocrine tumor; QD, once daily; QoL, quality of life; RCC, renal cell carcinoma; RP2D, recommended phase 2 dose; TID, three times daily; VEGF-TKI, vascular endothelial growth factor tyrosine kinase inhibitor; VHL, von Hippel-Lindau

Trial/NCT	Phase	Indication	Treatment plan	No of participants	Age group of participants	Results	Adverse events	Outcome/summary
LITESPARK-001 (MK-6482-001) NCT02974738 [29]	Phase I	Advanced solid tumors, including clear cell renal cell carcinoma	Belzutifan monotherapy (dose escalation to dose expansion)	Enrolled, 120; data available, 55	18 years and older (adult, older adult)	The data from 55 patients revealed ORR - 25% (14/55), MFU - 41.2 months	AE - 96%, among which the most significant were Grade 3 events reportedly anemia (24%) and hypoxia (13%)	RP2D-120 mg QD Comments - Demonstrated anticancer activity with manageable safety
LITESPARK-004 (MK-6482-004) NCT03401788 [2]	PHASE II	Von Hippel-Lindau (VHL) Disease-Associated Renal Cell Carcinoma (RCC) (with other VHL tumors)	Belzutifan monotherapy (120 mq QD dose)	Enrolled,50; data available, 50	18 years and older (adult, older adult)	In VHL-associated RCC, MFU-21.8 ORR-49% (CI 95%,36-62) (all partial responses). In other VHL tumors, such as a higher ORR of 77% in pancreatic lesions,30% in CNS hemangioblastomas, and 100% improvement in retinal hemangioblastomas	Low-grade adverse events comprising mainly anemia 90% and fatigue 66%	Comment - Data were generated for approval
LITESPARK-003 NCT03634540 [9]	Phase II	Advanced clear-cell renal cell carcinoma that was previously untreated (cohort 1) or previously treated with immunotherapy (cohort 2)	belzutifan 120 mg QD + cabozantinib 60 mg QD	Enrolled, 118, with 50 in Cohort 1	18 years and older (adult, older adult)	MFU-24.3 months ORR = 70% (35/50 patients, 95% CI 55-82) with 8% complete response and 62% partial response.	The most common Grade 3-4 adverse events were hypertension (12%), anemia (10%), and fatigue (8%). No treatment-related deaths reported	In treatment naïve patients treated with combination drugs revealed higher ORR, durable response with manageable toxicity.
LITESPARK-004 (MK-6482-004) NCT03401788 [35]	PHASE II (subset analysis)	VHL disease-associated RCC and other neoplasms with focus on VHL disease-associated pancreatic lesions [pancreatic neuroendocrine tumors (pNET) and serous cystadenomas]	Belzutifan monotherapy (120 mq QD dose)	Data available for 61	18 years and older (adult, older adult)	MFU-37.8 Pancreatic Lesions (all 61 patients) ORR = 84% (51/61 responders with 17 complete responses (CR)) pancreatic neuroendocrine tumors (pNETs) ORR = 91% (20/22 responders with 7 complete responses (CR))	18% patients experienced >1 Grade 3 AE	Demonstrating tumor shrinkage and activity in a pancreatic lesion
LITESPARK-005 NCT04195750 [23,36]	Phase III	Advanced ccRCC patients who had been previously treated with immune checkpoint and antiangiogenic therapies (ICI + VEGF-TKI)	Belzutifan 120 mg QD vs. 10 mg everolimus QD	Enrolled, 755; data available (belzutifan) 374 vs. everolimus 372)	18 years and older (adult, older adult)	1^st^ Interim MFU-18.4 months ⦁PFS in both groups -5.6 months ⦁24% of belzutifan-treated patients vs. 8.3% of everolimus-treated patients remained PF at 18 months (P = 0.002) ⦁Belzutifan ORR = 21.9% vs. Everolimus ORR = 3.5% with P < 0.001 → statistically significant superiority of belzutifan. MFU - 25.7 months OS-21.4 months with belzutifan vs. 18.1 months with everolimus HR for death -0.88, 95% CI 0.73-1.07, with P =0.2	Grade 3 AE occurred in 61.8% with belzutifan vs. 62.5% in everolimus. Grade 5 AE in 3.5% belzutifan vs. 5.3% in everolimus. Treatment discontinuation due to AE was 5.9% in belzutifan vs. 14.7% in everolimus.	Week 17 - Time to detoritation -physical functioning 19.3 in belzutifan vs. 13.8 months in everolimus with HR -0.93 -role functioning in belzutifan 12 months vs. 10.2 months in everolimus with HR -0.88 FKSI-DRS improved by least squares mean difference of 1.5 (95% CI 0.7-2.2) favoring belzutifan QLQ-C30global health/QoL improved by 6.4 (95% CI 3.2-9.6) in belzutifan in comparison to everolimus. Physical functioning and role functioning were similar, thus supporting label expansion improved response in belzutifan
LITESPARK-013 (NCT04489771) [37]	Phase II (Randomized dosed)	Patients with advanced ccRCC whose disease progressed after one to three prior systemic therapies, including an anti-PD-(L)1 regimen	Belzutifan 120 QD vs. higher dose/ alternative schedule	Enrolled, 154; data available - (120 mg- n = 76) vs. (200 mg - n =78)	18 years and older (adult, older adult)	MFU-20.1 Months (ORR) of 120 mg: 23.7% vs. 200 mg: 23.1%, with P = 0.5312 → No significant difference. PFS (hazard ratio (HR) 0.94, 95% CI 0.63-1.40) OS (medians not reached; HR 1.11, 95% CI 0.65-1.90)	Grade 3 or 4 AE with 35 patients (46.1%) in the 120 mg group vs. 36 patients (46.2%) in the 200 mg group.	Data revealed no significant difference between doses. Both doses demonstrate comparable antitumor activity and a constant safety profile, helping refine the dose toxicity relationship.
LITESPARK-015 (NCT04924075) [38]	Phase II global	Patients with locally advanced or metastatic pheochromocytoma or paraganglioma, VHL-associated tumors, and HIF-2α-altered solid tumors	Belzutifan monotherapy (120 mq QD dose)	Enrolled 322 Data available-72	12 years and older (Child, adult, older adult)	MFU-30.2 MONTHS ORR of 26% with 95% CI: 17%-38%. Disease control - 85% (95% CI, 74-92) MDR - 20.4 months. MPFS - 22.3 months. OS in 24 months was 76%.	99% of all participants reported AE with Grade 3 anemia in 22%, Serious treatment-related AE in 11%	Among 60 patients on baseline antihypertensive medication, 32 % reported 50% decrease in daily medicine dose for >6 months after initiating belzutifan
LITESPARK-018 NCT04846920 [39]	Phase I	Advanced ccRCC	Belzutifan alternative dosing or scheduling	Enrolled, 52; data available, 29	18 years and older (adult, older adult)	MFU-14.4 MONTHS 26 patients in arm A (160 mg BID n = 6; 160 mg TID n = 10; 200 mg TID n = 10) and 3 patients in arm B (120 mg QD) ORR-7% (2 partial responses)	100% AE with 99% having anemia. Grade 3-5 AE were anemia (48%) and hypoxia (31%). A grade 3 dose-related toxicity(hypoxia) occurred in each subgroup of arm A	Maximum tolerated dose not reached. Dose escalation and frequent dosing confirmed exposure response and safety profile.

**Table 2:** Ongoing clinical trials of belzutifan in renal cell carcinoma (as of December 12, 2025). Data compiled by authors from published clinical trials and regulatory sources [40-49]. AE, adverse events; AUC0-24, area under the plasma concentration-time curve from 0 to 24 hours; CBR, clinical benefit rate; CI, confidence interval; Cmax, maximum plasma concentration; CR, complete response; DCR, disease control rate; DFS, disease-free survival; DLT, dose-limiting toxicity; DOR, duration of response; MTD, maximum tolerated dose; ORR, objective response rate; OS, overall survival; PFS, progression-free survival; PR, partial response; PRO, patient-reported outcomes; Q6W, every 6 weeks; QoL, quality of life; RFS, recurrence-free survival; SD, stable disease; Tmax, time to maximum plasma concentration; TTR, time to response

Trial/NCT	Phase	Status	Enrolled participants	Age of the participants	Indication	Treatment given	Primary end points/outcome	Secondary end points/outcome
LITESPARK-011 NCT04586231 [40]	Phase III	Active, not recruiting	708	18 years and older (adult, older adult)	Patients with advanced RCC who have been previously treated with anti-PD-1/L1 therapy	Belzutifan + lenvatinib versus cabozantinib	PFS OS	ORR, DOR, safety, and tolerability
LITESPARK-012 NCT04736706 [41]	Phase III	Active, not recruiting	1,653	18 years and older (adult, older adult)	First line advanced cc RCC with no previous systemic treatments	Pembrolizumab + lenvatinib with vs. without belzutifan or quavonlimab (doublet vs. triplet)	PFS	ORR, DOR, safety, and tolerability. Comment: The study may support the possibility of triplet therapy by adding belzutifan to pembrolizumab + levothyroxine
LITESPARK-022 NCT05239728 [42]	Phase III adjuvant	Active, not recruiting	1,800	18 years and older (adult, older adult)	High-risk ccRCC after nephrectectomy	Belzutifan 120 mg orally once daily plus pembrolizumab 400 mg IV every 6 weeks (Q6W) vs. oral placebo plus pembrolizumab 400 mg IV Q6W	DFS - evaluating whether the combination improved DFS compared with pembrolizumab alone	Safety, disease recurrence, specific survival, patient reported outcome. Comment: Looking if the combination improved DFS vs. pembrolizumab alone
NCT07187779 [43]	Phase II	Not yet recruiting	Estimated 10	18 years and older (adult, older adult)	Localized ccRCC before nephrectomy surgery	Neoadjuvant belzutifan alone, pembrolizumab alone vs. combination before surgery	ORR, PRR	RFS, Safety/Tolerability, Quality of Life
LITESPARK-024 NCT05468697 [44]	Phase I/II	Active Not Recruiting	210	18 years and older (adult, older adult)	Patients with advanced ccRCC who progressed on or after receiving prior therapy	Belzutifan + palbociclib vs. belzutifan monotherapy	DLT, AE, ORR	CBR, DOR, PFS, OS, AE
NCT626518 KEYMAKER-U03- Substudy 03B [45]	Phase Ib/II	Active, not recruiting	370	18 years and older (adult, older adult)	Advanced second line plus (2L+) ccRCC	Belzutifan + lenvatinib (arm B5) ± other combinations	DLT, AE, ORR	DOR, PFS, OS, CBR
NCT04626479 [46]	Phase Ib/II	Active, not recruiting	400	18 years and older (adult, older adult)	Untreated first-line advanced ccRCC	Coformulation of vibostolimab/pembrolizumab + belzutifan	DLT, AE, ORR	DOR, PFS, OS, CBR
NCT06234605 [47]	Phase Ib	Recruiting	Estimated 80	18 years and older (adult, older adult)	Advanced solid tumors, including advanced or metastatic RCC with clear cell histology	HC-7366 + belzutifan	MTD and/or recommended Phase 2 dose (RP2D)	ORR, DOR, TTR, DCR, PFS, and OS
NCT05030506 [48]	Phase I	Active, not recruiting	45	18 years and older (adult, older adult)	Advanced ccRCC in the Chinese population	Belzutifan mono therapy or combination with lenvatinib +/- pembrolizumab	DLT, AE, AUC0-24, Cmax, Tmax	ORR, DOR, PFS, OS
NCT04627064 [49]	Phase I	Completed	11	18 years and older (adult, older adult)	Advanced RCC	Abemaciclib alone or abemaciclib belzutifan	ORR, MTD	AE, DOR, PFS, OS

**Table 3:** Ongoing clinical trials of belzutifan in non-renal cell carcinoma malignancies and rare disorders (as of December 12, 2025). Data compiled by authors from published clinical trials and regulatory sources [50-55]. AE, adverse events; AUC, area under the plasma concentration–time curve; CBR, clinical benefit rate; Cmax, maximum plasma concentration; DCR, disease control rate; DFS, disease-free survival; DLT, dose-limiting toxicity; DOR, duration of response; ER+, estrogen receptor positive; HER2−, human epidermal growth factor receptor 2 negative; MTD, maximum tolerated dose; ORR, objective response rate; OS, overall survival; PFS, progression-free survival; QoL, quality of life; rPFS, radiographic progression-free survival; t½, terminal half-life; Tmax, time to maximum plasma concentration

Trial/NCT	Phase	Status	Enrolled participants	Age of the participants	Indication	Treatment	Primary end points/outcome	Secondary end points/outcome
NCT04976634 [50]	Phase II	Active not recruiting	730	18 years and older (adult, older adult)	Multiple solid tumors, including hepatocellular carcinoma, colorectal cancer, pancreatic ductal adenocarcinoma, biliary tract cancer, endometrial cancer, and esophageal squamous cell carcinoma.	Pembrolizumab + lenvatinib + belzutifan (triplet)	DLT, AE, ORR	DOR, DCR, PFS, OS
NCT06428396 [51]	Phase II	Recruiting	Estimated 120	18 years and older (adult, older adult)	ER+/HER2- advanced unresectable or metastatic breast cancer that has progressed on endocrine therapy	Belzutifan + fulvestrant vs everolimus + fulvestrant or exemestane	PFS	PFS, ORR, and safety
NCT06677190 [52]	Phase II	Recruiting	Estimated 32	18 years and older (adult, older adult)	Persistent or relapsed clear cell ovarian carcinoma, as well as clear cell carcinoma originating from other gynecologic organs	Belzutifan monotherapy	PFS, ORR	OS, PFS, CBR, Grade 4 treatment-related toxicity rate
NCT02861573 [53]	Phase Ib/II	Recruiting	1200	18 years and older (adult, older adult)	Metastatic castration-resistant prostate cancer (post-docetaxel)	Arm J1: belzutifan; Arm J2: pembrolizumab + belzutifan	AEs, ORR	DCR, OS, DOR, rPFS, composite response rate
NCT04994522 [54]	Phase I	Completed	14	18 years and older (adult, older adult)	ESRD before or after hemodialysis	Belzutifan monotherapy 120 mg dose	AUC, Cmax, Tmax, apparent terminal half-life (t½)	Dialysis clearance, AE
NCT04995484 [55]	Phase I	Completed	17	18 years and older (adult, older adult)	Participants with moderate hepatic impairment	Belzutifan 80 mg monotherapy	AUC, Cmax, Tmax	AE

Safety profile of belzutifan 

*Adverse Effects and Special Safety Considerations*

Because belzutifan is administered over extended treatment durations, close monitoring for adverse events that could necessitate dose interruption or discontinuation is essential [9]. Safety data from the LITESPARK-004 study showed that most adverse events were grade 1 or 2 and occurred in 20 patients (33%). Grade 3 treatment-related adverse events were reported in 9 patients (15%). During the first 13 weeks of treatment, all patients experienced a decrease in hemoglobin levels. One case of grade 3 transient hypoxia resolved following a dose reduction to 80 mg without the need for supplemental oxygen. Serious adverse events, including anaphylaxis, retinal detachment, and central retinal vein occlusion, were each reported in one patient. Overall, this study demonstrated that belzutifan was not associated with frequent adverse events leading to treatment discontinuation, although common side effects warrant continued monitoring and follow-up [9].

The adverse event profile of belzutifan is most frequently characterized by fatigue, anemia, and hypoxia. Belzutifan may also affect fertility and requires caution when used during pregnancy. These adverse effects are related to inhibition of HIF-2α-regulated targets, including EPO, PDGFR, GLUT1, and VEGF. Suppression of transcriptional activity in these pathways contributes to anemia, hypoxia, and fatigue. Additional commonly reported adverse effects include increased serum creatinine, headache, dizziness, hyperglycemia, and nausea [9].

Anemia

Anemia represents the most commonly observed toxicity during belzutifan therapy. This effect is driven by dose- and exposure-dependent suppression of EPO signaling and serves as an indicator of pharmacologic target engagement [21]. According to FDA prescribing information, a mean 60% reduction in EPO levels occurs within the first two weeks of continued dosing, with gradual recovery toward baseline by approximately 12 weeks of treatment. In clinical monitoring, a hemoglobin level below 9.0 g/dL is an indication to temporarily discontinue belzutifan until recovery. Although erythropoiesis-stimulating agents (ESAs) may theoretically mitigate anemia, their use is limited because some ccRCCs express EPO receptors, potentially promoting tumor progression. Moreover, ESA use in patients receiving myelosuppressive chemotherapy has been associated with increased mortality and cardiovascular risk. Accordingly, routine use of ESAs is discouraged, with anemia management relying instead on clinical monitoring, dose adjustment, and transfusion support when indicated [21].

Hypoxia

Hypoxia is another commonly observed adverse effect and warrants baseline and ongoing monitoring with pulse oximetry during treatment. The target oxygen saturation is above 88%, and values below this threshold indicate the need to withhold treatment until resolution. The underlying mechanism of hypoxia is less clearly defined than that of anemia but may involve reduced oxygen-carrying capacity due to anemia and possible hypoventilation. When hypoxia or other adverse effects occur, dose reduction is generally recommended, initially to 80 mg orally once daily, followed by a reduction to 40 mg if symptoms persist. Permanent discontinuation is advised if adverse effects do not resolve despite dose modification [21].

Fertility and Pregnancy Considerations

There is currently no clear evidence regarding the effects of belzutifan on fertility or pregnancy in humans. However, animal studies have demonstrated irreversible testicular atrophy and hypospermia in male rats. During fetal organogenesis, pregnant rats exhibited embryo-fetal lethality, reduced fetal body weight, rib malformations, and impaired skeletal ossification. No reproductive toxicity was observed in female rats. In light of these observations, patients should receive counseling regarding potential reproductive risks, and pregnancy status should be confirmed before initiating therapy. Belzutifan may reduce the effectiveness of hormonal contraceptives, potentially leading to unexplained bleeding or contraceptive failure. Although direct harm has not been established, breastfeeding is not recommended until at least one week after the final dose of belzutifan [21].

Rare, Serious, and Emerging Safety Signals

In addition to the more common adverse events, several less frequently reported but potentially serious safety signals have been observed and warrant continued clinical vigilance and post-marketing surveillance. In the LITESPARK-004 study, a case of grade 2 intracranial hemorrhage occurred in a patient receiving anticoagulation therapy and was considered unrelated to belzutifan. However, post-marketing reports have described hemorrhagic events in patients with VHL-associated CNS hemangioblastomas, including two cases occurring within days of belzutifan initiation and one case of spinal hemangioblastoma hemorrhage in a patient treated for ccRCC, resulting in transient paralysis with subsequent recovery. These observations highlight the importance of systematic international collection of adverse event data for belzutifan [56].

Cardiac toxicity is a recognized complication of several anticancer therapies, particularly immune checkpoint inhibitors. A reported case of biopsy-proven myocarditis occurred in a patient with metastatic RCC previously treated with pembrolizumab, followed by lenvatinib and belzutifan. Given the established association between pembrolizumab and immune-mediated myocarditis, this event was considered most consistent with a delayed immune-related adverse effect occurring months after treatment discontinuation. Nevertheless, as myocarditis developed during combined therapy with belzutifan and lenvatinib, a contributory role of belzutifan cannot be definitively excluded, underscoring the need for continued cardiac safety evaluation in patients receiving HIF-2α inhibitors [57].

Drug Interactions and Organ Function Considerations

Concomitant use of belzutifan with UGT2B17 or CYP2C19 inhibitors increases belzutifan plasma concentrations and may heighten the risk of adverse effects, necessitating close monitoring of hemoglobin levels and oxygen saturation. Conversely, belzutifan may reduce exposure to CYP3A4 substrates, potentially diminishing the therapeutic effects of commonly prescribed medications such as statins, calcium channel blockers, benzodiazepines, corticosteroids, immunosuppressants, antiretroviral agents, and opioids. Given the widespread use of these agents in oncology populations, careful attention to potential drug-drug interactions is required [21].

Dose modification is not recommended for patients with mild to moderate renal or hepatic impairment. However, FDA prescribing information indicates that no formal studies have evaluated belzutifan dosing in patients with severe renal or hepatic dysfunction [21]. To address this knowledge gap, a dedicated clinical pharmacology study (NCT04994522) has been initiated to assess the pharmacokinetics and safety of belzutifan in patients with end-stage renal disease, including evaluations before and after hemodialysis, compared with healthy controls. Until results from this study are available, use of belzutifan in dialysis-dependent patients should rely on cautious clinical monitoring rather than empiric dose adjustment [21].

QoL, tolerability, and physician perspectives on belzutifan

Available PROs and QoL Data

Currently, prospective PRO and health-related QoL data specific to belzutifan are limited.

PROs from the phase III LITESPARK-005 study were evaluated in patients with advanced RCC randomized to receive belzutifan or everolimus, with PRO data collected across both treatment groups. Completion rates for PRO questionnaires were high at baseline and remained adequate through week 17, with more than half of participants continuing to provide evaluable responses [36].

During the initial 17 weeks of therapy, patient-reported symptom burden and QoL measures favored belzutifan over everolimus. Least-squares mean differences favored belzutifan for the Functional Assessment of Cancer Therapy-Kidney Symptom Index Disease-Related Symptoms subscale (FKSI-DRS) (+1.5; 95% CI, 0.7-2.2) and the EORTC QLQ-C30 global health score (6.4; 95% CI, 3.2-9.6) [36].

No clinically meaningful differences were observed in physical or role functioning between treatment groups, and time to deterioration was comparable for physical functioning (19.3 vs. 13.8 months; HR, 0.93) and role functioning (12.0 vs. 10.2 months; HR, 0.88). These findings suggest that belzutifan preserves QoL while providing clinical benefit compared with everolimus in patients with advanced RCC [36].

Indirect QoL Signals From Safety and Tolerability

In the absence of extensive direct PRO data, belzutifan’s safety and tolerability profile provides indirect evidence regarding its effects on patient QoL. Several features of belzutifan may contribute to patient convenience, including oral administration, which eliminates the logistical burden associated with infusion-based therapies. Additionally, belzutifan has a lower risk of immune-related adverse events compared with immune checkpoint inhibitors. The absence of cumulative neuropathy or hand-foot syndrome, which is commonly associated with VEGF-targeted TKIs, may also improve tolerability [9,19,23,28].

The common side effects of belzutifan, such as anemia, fatigue, and hypoxia, can affect QoL. These adverse effects are typically predictable, can be easily monitored in routine clinical practice, and are usually manageable with dose adjustments and supportive care. As a result, belzutifan may allow sustained treatment while preserving functional well-being in heavily pretreated patients with advanced RCC [9,19,23,28].

*QoL* *Considerations in VHL*

Although formal PRO measures were not incorporated into pivotal clinical trials, belzutifan has substantial QoL relevance in patients with VHL disease. VHL is characterized by a lifelong predisposition to multifocal renal, CNS, and pancreatic tumors, often necessitating repeated surgical interventions that cumulatively increase morbidity and risk of organ dysfunction [9,58].

Belzutifan provides a systemic therapeutic option capable of inducing tumor regression or sustained disease stabilization across multiple VHL-associated lesions, thereby reducing reliance on serial surgical procedures. In this context, QoL benefit is inferred indirectly through prolonged disease control, preservation of organ function, and avoidance or delay of invasive interventions. Accordingly, the impact of belzutifan in VHL disease is best conceptualized as a reduction in cumulative disease and treatment burden rather than as an improvement in standardized symptom-based measurements [9,58].

Future Directions for QoL Assessment

As the clinical use of belzutifan continues to expand, important gaps remain in the evaluation of patient-centered outcomes and QoL. Future clinical trials should prioritize the incorporation of disease-specific PRO measures assessing fatigue, physical functioning, and dyspnea as predefined endpoints. Comparative QoL analyses against commonly used systemic therapies, including immune checkpoint inhibitors and VEGF-targeted agents, would be particularly informative given their distinct toxicity profiles and cumulative treatment burdens. In addition, real-world studies and longitudinal registries may provide valuable insights into long-term QoL outcomes, particularly in patients with VHL disease [9,59,60].

Physician Perspective, Tolerability, and Clinical Acceptance of Belzutifan

Among treating clinicians, belzutifan is increasingly viewed as a feasible long-term systemic therapy, supported by its targeted mechanism, once-daily oral dosing schedule, and predictable toxicity profile. Among specialists involved in the management of VHL-associated renal malignancies, 98% anticipated a meaningful shift in clinical practice following its approval, and approximately 76% expressed a preference for continuous long-term therapy over intermittent or post-resection approaches, reflecting substantial clinical confidence [9].

Notable subspecialty-based differences were observed in prescribing attitudes, with a higher willingness to prescribe belzutifan among medical oncologists (approximately 91%) compared with urologists (38%; *P* = 0.02). These differences likely reflect variation in therapeutic orientation and familiarity with prolonged systemic treatment. Divergent opinions were also reported regarding management strategies, including early initiation versus active surveillance for small renal masses (<3 cm) and approaches to managing tumor growth during ongoing belzutifan therapy [9].

Acceptance of belzutifan among specialists is driven in part by its predictable adverse effects, which are primarily based on its mechanism of action and can be managed through monitoring and dose adjustments. This profile is particularly advantageous in chronic conditions such as VHL disease, where preservation of organ function and minimization of intervention are central to patient care. Overall, evidence from routine clinical use and physician surveys supports increasing confidence in belzutifan as a patient-centered therapeutic option, while pointing to the need for additional research to better define clinical practice patterns and QoL perceptions across oncology subspecialties [9,60,61].

Other HIF-2α inhibitors in clinical development

The clinical validation of belzutifan has established HIF-2α as a viable therapeutic target and prompted the development of additional inhibitors within this pathway. Several next-generation agents are now in early clinical evaluation, aiming to refine HIF-2α inhibition and expand its therapeutic scope.

NKT2152

NKT2152 was designed as a next-generation HIF-2α inhibitor with the objective of achieving more sustained pathway inhibition compared with earlier agents. Clinical evaluation of NKT2152 began in an open-label phase I/II study assessing once-daily oral monotherapy in patients with advanced ccRcc. The phase I component emphasized dose optimization, while the phase II component evaluated antitumor activity using objective response rate as the primary endpoint, alongside secondary assessments of safety, pharmacodynamics, progression-free survival, duration of response, disease control, and exploratory biomarkers [62].

Along with monotherapy, combination strategies were pursued to investigate the potential synergistic effects in advanced or metastatic ccRCC. This included phase 2 combination including dual therapy with palbociclib and triple therapy with palbociclib and sasanlimab were initiated to explore potential synergistic activity, in patients with advanced or metastatic ccRCC. Despite early signals of clinical activity in heavily pretreated patients, all ongoing NKT2152 clinical trials were ultimately terminated for strategic portfolio reasons (NCT05119335, NCT05935748) [62].

Casdatifan (AB521)

Casdatifan is an investigational HIF-2α inhibitor under development to interfere with hypoxia-driven oncogenic signaling pathways. It is being developed and clinically evaluated with a primary focus on ccRCC while exploring its role in other solid tumors. Casdatifan is currently under clinical investigation and has not been approved by the FDA [62].

Clinical development of casdatifan includes the ARC-20 study, an open-label phase I trial enrolling patients with ccRCC and selected hypoxia-driven solid tumors. The study evaluates dose escalation and expansion cohorts of casdatifan administered as monotherapy, with primary outcomes including safety, tolerability, and determination of dose-limiting toxicities. Secondary outcomes being evaluated include objective response, pharmacokinetics, and systemic drug exposure (NCT05536141) [62].

In parallel with monotherapy optimization, casdatifan is being studied in combination regimens with the VEGF inhibitor cabozantinib within the ARC-20 program (NCT07011719) and with zanzalintinib in the phase Ib/II STELLAR-009 study. However, advancement of the STELLAR-009 study was placed on hold due to commercial considerations (NCT06191796). Additional combination strategies are planned, including a phase Ib trial evaluating casdatifan with volrustomig (NCT07000149). These initiatives position casdatifan as a promising next-generation HIF-2α inhibitor with potential applications across both monotherapy and combination treatment settings [62].

DFF332

DFF332 has been evaluated as an HIF-2α inhibitor in an open-label phase I/Ib clinical study involving patients with advanced ccRCC. The trial aims to explore the outcomes of safety, tolerability, pharmacokinetics, and preliminary antitumor activity of the drug [62,63].

Phase I evaluation demonstrated a generally favorable safety profile with consistent tolerability across dose levels. Pharmacodynamic analyses showed evidence of HIF-2α engagement, reflected by dose-dependent EPO production. Among approximately 40 treated patients, with a median treatment duration of 12.1 weeks, low-grade treatment-related adverse events were reported in about 63% of participants. Although the trial showed an overall tolerable safety profile, premature cessation of dose escalation limited definitive conclusions regarding optimal dosing and efficacy. Further research is needed to define the therapeutic potential of DFF332, including the optimal dose, potential benefits, and its role as combination therapy in hypoxia-driven ccRCC (NCT04895748) [62,63].

Future directions for belzutifan: biomarkers, resistance, and first-line integration

Belzutifan’s short-term future is shaped by three interrelated goals: identifying patients with the greatest likelihood of response, optimizing therapeutic combinations or sequencing approaches, and addressing on-target adverse effects that limit its use in the first-line setting. From a biomarker perspective, dependence on the VHL/HIF-2α pathway represents the most biologically plausible predictor of response. VHL inactivation is a defining molecular driver of ccRCC and underpins belzutifan’s activity in VHL disease, where durable multisite tumor responses reflect a tumor phenotype that is particularly susceptible to HIF-2α inhibition [5,9].

A complementary biomarker framework for belzutifan involves mechanisms of primary and acquired resistance. Resistance has been associated with gatekeeper mutations in HIF-2α that impair allosteric inhibitor binding, as well as adaptive molecular changes that evolve under selective pressure from HIF-2α inhibition. This evolving resistance landscape supports the use of serial ctDNA or next-generation sequencing (NGS) analyses to identify emerging HIF2A variants and reinforces the need for next-generation HIF-2-targeting approaches and combination strategies to mitigate resistance [23,64].

Despite the encouraging activity, belzutifan’s integration into the first-line treatment of metastatic ccRCC remains limited by the current evidence base. The strongest phase III support for belzutifan monotherapy comes from studies conducted after prior ICI and VEGF-targeted therapy, confirming its efficacy in later-line settings without establishing an advantage over current first-line regimens [23]. Adoption in the first-line setting is further challenged by the availability of multiple highly effective ICI-based doublets that define current standard-of-care regimens, necessitating clear evidence of incremental benefit in efficacy, tolerability, or other clinically meaningful domains. Moreover, belzutifan’s on-target toxicities, such as anemia and hypoxia, although generally manageable, may be perceived as less acceptable in the first-line setting, where alternative therapies offer different safety profiles. This may limit broader adoption until optimal management strategies and predictive biomarkers are established [9,23].

A plausible route for belzutifan to gain first-line relevance is through rational combination or triplet regimens validated in comparative clinical trials. Initial first-line experience combining belzutifan with cabozantinib has demonstrated favorable activity in untreated advanced ccRCC. These findings suggest that pairing HIF-2α inhibition with VEGF/TKI therapy may broaden antitumor responses and help overcome the biological limitations of monotherapy [27].

Ongoing phase III trials are exploring the integration of belzutifan into established first-line regimens for advanced RCC. LITESPARK-012 is assessing whether the addition of belzutifan to the pembrolizumab/lenvatinib backbone can improve therapeutic outcomes. Results of this trial will help clarify the potential role of HIF-2α inhibition in the first-line treatment landscape [12].

## Conclusions

Belzutifan marks a major advancement in the therapeutic modulation of hypoxia signaling mechanisms, converting long-standing biological understanding of the VHL/HIF axis into clinical benefit for patients with VHL-associated tumors and advanced ccRCC.

Belzutifan demonstrates durable antitumor activity with a predictable, mechanism-driven safety profile. The most common adverse effects are anemia, hypoxia, and fatigue, which are manageable with routine monitoring and supportive care. The convenience of oral dosing and absence of cumulative vascular or immune-mediated toxicities distinguish it from VEGF-TKIs and immune checkpoint inhibitors, making it an attractive option for long-term treatment in chronic conditions such as VHL disease. Importantly, emerging patient-reported outcomes suggest preservation of quality of life, supporting its overall clinical acceptability. Belzutifan’s development exemplifies a shift toward biologically informed, pathway-driven precision oncology, where targeting core disease dependencies rather than empiric cytotoxic or anti-angiogenic approaches reshapes the conceptual framework for targeted therapy development in solid tumors.

Expansion of belzutifan into the first-line treatment of metastatic ccRCC will depend on addressing unresolved gaps in predictive biomarker validation, limited characterization of resistance pathways, and uncertainty regarding ideal sequencing or combination regimens. Although VHL/HIF-2α pathway dependence offers a framework for patient selection, prospective confirmation is needed. In addition, mechanism-driven target toxicities such as anemia and hypoxia must be carefully considered as belzutifan is evaluated in earlier-line and combination settings.

Overall, the clinical development of belzutifan has confirmed HIF-2α as a viable target, stimulating new pathway-based treatment strategies and combination approaches. Ongoing investigation will be critical to clarify its optimal clinical role and to fully realize its potential as a patient-centered therapeutic approach in cancers driven by hypoxia.
